# Soft threshold partial least squares predicts the survival fraction of malignant glioma cells against different concentrations of methotrexate’s derivatives

**DOI:** 10.1038/s41598-021-97891-3

**Published:** 2021-09-21

**Authors:** Tahir Mehmood, Mudassir Iqbal

**Affiliations:** School of Natural Sciences (SNS), National University of Sciences and Technology (NUST), Islamabad, Pakistan

**Keywords:** Biochemistry, Medical research, Statistics

## Abstract

Chemotherapy appeared to be a significant advancement in cancer research, with fewer side effects. Methotrexate (MTX) is a widely used anticancer drug with strong activity but serious side effects. Several MTX derivatives have been reported, with modifications at various sites to reduce side effects and increase efficacy. The current study uses FTIR spectroscopy to predict the survival fraction of human malignant glioma U87 (MG-U87) cell lines against MTX derivatives. Together with Parent MTX several aldehydes viz. Benzaldehyde, Chlorobenzaldehyde, 2-Chlorobenzaldehyde, 3-Nitrobenzaldehyde, 5-Chloro-2-hydroxybenz-aldehyde, 2-Hydroxy-5-Nitrobenzaldehyde, 2-Thiocarboxyaldehyde, Trans-2-pentenal, and Glutaraldehyde are treated with MTX to obtain MTX derivatives. The prediction of survival fraction of malignant glioma cells is carried out by Lasso, Elastic net and Soft PLS at different concentration levels of synthesized derivatives, including 400 μM, 200 μM, 100 μM, 50 μM, 25 μM and 12.5 μM. The cross-validated prediction error is minimised to optimise spectral wavelength selection and model parameters. It appears that the RMSE computed from test data is significantly varying with the change of models (p = 0.012), with the change of concentrations levels (p $\le 0.001$) and with the change of combination of models and concentration level (p $\le 0.001$). StPLS outperforms in predicting survival fraction of glioma cells at the concentration level 50 μM, 100 μM and 400 μM respectively with relative RMSE = 0.1,0.14 and 0.55. Lasso outperforms at the concentration level 12.5 μM, and 200 μM respectively with relative RMSE = 0.4 and 0.14. Elastic net outperforms at the concentration level 25 μM with relative RMSE = 0.8. Consistently appeared influential wavelength identifies the influential functional compounds which best predicts the survival fraction. Hence FTIR appears potential candidate for estimating survival fraction of MTX derivatives.

## Introduction

Methotrexate (MTX) is certainly one of the broadly used antimetabolites in cancer chemotherapy that terminates DNA replication mainly by snooping with thymidylate synthesis pathway^1,2^. It encompasses several cancer types for example leukemia, gestational trophoblastic disease, lung cancer, breast cancer, osteosarcoma and lymphoma^3^. Different levels of MTX’s dosages are recommended for different types of diseases. For instance, low dose MTX treats Rheumatoid arthritis and other types of inflammatory diseases^4^, and high doses MTX is used for intraocular lymphoma^5^. Hence relevant concentrations of MTX are of importance. Together with the wide applications of MTX, it has some drawbacks as it induces life-threatening side effects on sensitive organs. Specific side effects of MTX include diarrhea, feeling weak, cough, increased chances of infection, reduced counts of white blood cell, Within mouth skin deterioration, liver failure, lung disease, lymphoma, and extreme skin rash^6^.

Modification in MTX structure enhances its anticancer activity^7^ and is expected to minimizes its side effects. MTX structure has several sites, where synthesized modifications in these sites results in MTX derivatives^8^. One possible way to measure the MTX and its derivatives’ biological effect is to measure the cancer cell survival fraction. It reflects the cancer cells that have retained their reproductive capacity after applying the cancer therapy^9^. This is customary presented through the survival curve, which describes the link between the absorbed dose and the fraction of cells that survive.

Cancer medications are believed to interact with cell metabolism by many pathways. For characterization of MTX derivatives Fourier Transformation Infrared (FTIR) spectroscopy is potential candidate which is low in cost and fast in processing, which is sensitive to all kinds of molecules present in cells, hence can provide unique and accurate fingerprints of samples. For example, FTIR has been used to predict the migration of glioma cell lines in vitro^10^. FTIR characterize metabolic difference of cardiotonic steroid family^11^, The subtoxic doses of gemcitabine, a cancer drug, can be monitored using FTIR spectroscopy^12^, it can model the metabolism disruption track cancer cells^13,14^, it can diagnose arthritis(rheumatoid) through serum^15^, it can recognize most of the cell types found in melanoma cancer^16^, it can discriminate the anticancer medication reference to their mode of operation for prostate cancer cells^17^, and it can provide label-free surveillance of therapeutic medicines that is Busulfan and Methotrexate in human serum^18^.

Chemometric analyses are mostly based on widely used partial least squares (PLS) regression. PLS is being extensively used for drug concentration predication, survival and fitness response predication^14–19^. Soft thresholding PLS (StPLS) is a potential variant adds the influential wavelength selection together with response prediction^20^. Together with PLS, elastic net (Elnet) and least absolute shrinkage and selection operator (Lasso ) are also in practice for modeling cancer cell functions^21,22^. These algorithms implements cyclical coordinate descent for computing the regularization path. These approaches have the inner ability to integrate knowledge from all kinds of molecules found in cells and create a spectral signature specific to a cell group. As a result, using FTIR spectroscopy, StPLS, Lasso, and Elnet can be used to predict the survival fraction of MTX derivatives against human malignant glioma U87 (MG-U87) cell lines. These methods work in two steps: model construction and validation.

In the current study, FTIR spectroscopy on human malignant glioma U87 (MG-U87) cell lines reveals that MTX derivatives develop a reproducible FTIR spectral signature at various concentrations for methotrexate’s unique inhibition of DNA synthesis.

## Methods

### Material

In this study together with Parent MTX schiff base MTX derivatives with target compounds several aldehydes viz. Benzaldehyde, Chlorobenzaldehyde, 2-Chlorobenzaldehyde, 3-Nitrobenzaldehyde, 5-Chloro-2-hydroxybenz-aldehyde, 2-Hydroxy-5-Nitrobenzaldehyde, 2-Thiocarboxyaldehyde, Trans-2-pentenal, and Glutaraldehyde are considered. MTX is a commonly used drug for various types of cancer. All the new derivatives were synthesized for the first time in study^23^. These cell lines were used to check the initial response as anticancer potential. Initial response can be checked on any cell lines. All solvents were of analytical grade and dried solvents were used in reaction scheme. All reactions were carried out in inert atmosphere of nitrogen. By using the rotary evaporator R-210 solvents were dried. Thin-layer chromatography analytic was conducted on Baker 250F and Whatman MK6F silica gel plates. By using TLC on silica gel plates reaction products were purified. By using UV lamp chemical reactions were visualized. The melting point of reactant and product was determined. These MTX derivatives were dissolved in dry ethanol in round bottom flask, NaOH and given aldehyde was added and reaction mixture was refluxed until TLC indicated disappearance of aldehyde. After completion of reaction pH was adjusted to 5, precipitate was washed with hexane and dried.

### Evaluation for anticancer activities

U87 cell lines were used to check the initial response as anticancer potential. These cell lines were imported from Merck, US supplier. Initial response can be checked on any cell lines. The established method for identification of the survival fraction of U87 is reported^23^. To evaluate the anticancer effect of all micro molar MTX derivatives, the growth inhibition assays on MG-U87 cell lines was observed over different MTX-derivative’s incubation times using incremental doses from 12.5 to 400 μM. The human malignant glioma U87 (MG-U87) cell lines were cultured in Dulbecco’s Modified Eagle’s Medium/F12 in 1:1 concentration, with addition of $$ L$$-Glutamine and sodium bicarbonate (2.438 g/L). The media was added with 10% fetal bovine serum, 1% Penicillin/Streptomycin, 0.5% Fungizone (Invitrogen, USA) and grown at 37$$^\circ $$C under 5% CO$$_2$$ supply. Following 80% confluency, the MG-U87 cell lines were seeded in 96 well plates at concentration of 2.5 $$\times $$ 10^4^ (200 μL/well) and were allowed to grow for 24 h at 37 $$^\circ $$C in 5% CO$$_2$$ supply. Meanwhile, drug dosages of methotrexate (MTX) and its derivatives were prepared in DMSO. After 24 h of growth, the cells were processed in triplicates along with several concentrations of MXT and its derivatives at dose of 0 μM (untreated control), 12.5 μM, 25 μM, 50 μM, 100 μM, 200 μM and 400 μM. The untreated cells in triplicates were considered as control. The drug treatment was conducted for 24 h to determine dose concentration that can inhibit 70% or 50% growth of the cells (IC70 or IC50 value). Following 1 day of drug treatment, the cells were fixed with 4% formaldehyde (Scharlab S.L, Spain) for 10 min that was followed by staining with 0.1% crystal violet (BioM labs, USA). After staining the 96 wells plates, each well was washed with distilled water and 100 μL of acetic acid was included to each well. The absorbance was measured at 630 nm wavelength through an ELISA plate reader (Z32HK: Germany). From, the growth inhibition assays, survival fraction of each MTX derivatives at different concentration levels is constituted in response matrix $$Y_{10 \times 6}$$.

### Spectroscopic experiment of MTX derivatives

Attenuated Total Reflection-Fourier Transformation Infrared (ATR-FTIR) spectrometer ALPHA 200488 which covers mid-IR (4000–550 cm$^{-1}$ wavenumbers was used. ATR-FTIR was maintained with UATR Diamond ATR (Single Reflection) and high linear room temperature detector. For each MTX derivative 10 scans were goatherd with 1 $cm/s$ scan speed and 4 $cm^{-1}$ resolution. Before each measurement background spectra against each MTX derivative was obtained. The spectrum obtained from this spectroscopic experiment of MTX derivatives were used to contract the data matrix $X_{10 \times 1676}$.

### Baseline correction

FTIR spectra includes linear or non-linear residuals from spectroscopic experiment results in non flat base line of spectra. For statistical modeling and analysis of spectral based data set flat line at zero is required^24^. For this use of baseline correction methods is in practice. For this asymmetric least squares (ALS)^25^ is considered. ALS is based on least squares algorithm which weights explanatory variables with positive differences. For smoothing $2^{nd}$ derivative restriction is incorporated through $S=\sum w_i (x_i- b_i)^2 + \lambda \sum (\Delta ^2b_i)$

where $b_i$ presents the estimated baseline $x_i$ presents the original spectrum, $w_i$ presents the asymmetric residual weights and $\Delta ^2$ presents second derivative of computed baseline. ALS is based on two parameters smoothing and weight denoted by $\lambda$ and $w$. For optimal estimation of these parameters cross validation procedure introduced in^24^ is used. For this several levels of these parameters are used. For each level of the MTX derivative’s spectra baseline is corrected and is used for further analysis (Supplementary Information).

### Predicting the survival fraction

Predicting the survival fraction of MTX’s derivatives through FTIR spectral data results in multivariate data with much much larger number of wavenumbers that is explanatory variables compared to the number of samples. Multivariate methods are considered as potential candidate^26,27^ for modeling multivariate data. For prediction purpose two streams exists, one uses iterative loading based procedure called partial least squares (PLS1) and others are based on penalized liner models includes elastic net (Elnet) and least absolute shrinkage and selection operator (Lasso )^28^. Soft thresholding (PLS1) (StPLS) is a potential variant adds the influential wavelength selection together with response prediction^20^. In order to improve prediction and interpretability, these methods use variable selection and regularisation. As a result, StPLS, Lasso, and Elnet are used in this study to predict the survival fraction of MTX derivatives against human malignant glioma U87 (MG-U87) cell lines using FTIR spectroscopy.

#### Soft-thresholding partial least squares (StPLS)

In basic PLS algorithm^26^ the covariance between response $y$ that is survival fraction of MTX derivatives and the explanatory variables’ linear combination $X$ that is MTX derivatives spectra are optimized through iterative components has

$$\begin{aligned} max Cov(p^t_h X , q^t_h y)\end{aligned}$$

where $p_h$ and $q_h$ are the $h^{th}$ X- and y- loadings respectively. In multivariate data sets larger number of explanatory variables compared to available sample size is very obvious. This scenario can increase the variation of estimated PLS coefficients^29,30^, hence motivates for variable selection. For variable selection in PLS soft-thresholding step in the PLS (StPLS) algorithm is introduced^20^. In each iteration components of StPLS loading-weights are computed as:

1. Scaling: $${w}_h \leftarrow {w}_h/\max _j |w_{h,j}|$$, where $$j=1,\ldots ,p$$
2. Soft-thresholding: $w_{h,j} \leftarrow \text{ sign }(w_{h,j})(|w_{h,j}|-\delta )_+$, where some $\delta \in [0,1\rangle$ and $(\ldots )_+$ indicates $\max (0,\ldots )$
3. Normalizing: $${w}_h \leftarrow {w}_h/\Vert {w}_h\Vert $$

The shrinkage parameter $\delta \in [0,1)$ explains the level of shareholding in StPLS this means larger $\delta$ will results in fewer selected set of variables. The optimal level of $\delta$ is determined by cross validation^20^.

### Penalized model

An alternative to iterative modeling procedure (PLS based) are penalized models. In this regard elastic net (Elnet) and least absolute shrinkage and selection operator (Lasso )^28^ are potential candidates. These model implements the coordinate descent algorithm with certain penalty parameter called $\theta$. For computational purpose several levels are considered and optimal choice of these penalty parameter is obtained through cross validation. Here the multivariate regression coefficient $beta$ is penalized through the partial log-likelihood function defined by

1 $$\begin{aligned} L(\varvec{\beta })- \sum _{j=1}^p p_{\theta ^*}(|\beta _j|) \end{aligned}$$

where $L(\varvec{\beta })$ denotes the partial log-likelihood for $n$ samples. $p_\theta (|\cdot |)$ presents the penalty function.

In lasso^31^ $L_1$-penalized model is used, which is defined as

2 $$\begin{aligned} p_\theta (|\beta |)=\theta | \beta | \end{aligned}$$

An alternative to $L_1$ penalty is $L_2$ penalty which shrinks some of the regression coefficients $\beta$, where $L_2$ penalty is

3 $$\begin{aligned} p_\theta (|\beta |)=\theta \beta ^2 \end{aligned}$$

In elastic net (Elnet)^32^ the mixture of both $L_1$ and $L_2$ penalty is used as

4 $$\begin{aligned} p_{\theta _1,\theta _2}(|\beta |)=\theta _1 | \beta | + \theta _2 \beta ^2. \end{aligned}$$

Both Lasso and Elnet do the variable selection by equating non significant variable’s coefficients to zero. Here $\theta _1$ is weight for $L_1$ penalty and $\theta _1$is weight for $L_2$ penalty, moreover $\theta _1 +\theta _2=1$. Small $\theta _2$ level will result large number of variables and vice versa.

## Results

**Figure 1:**
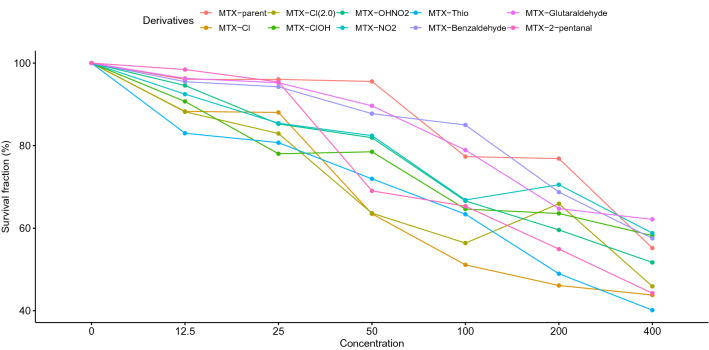
At various concentrations, the survival fraction of these MTX derivatives against human malignant glioma U87 (MG-U87) cell lines is presented.

In current study 1 MTX parent and 9 MTX derivatives are considered, which include Schiff base derivatives MTX with Benzaledehyde, Chlorobenzaldehyde, 2-Chlorobenzaldehyde, 3-Nitrobenzaldehyde, 5-Chloro-2-hydroxybenz-aldehyde, 2-Hydroxy-5-Nitrobenzaldehyde, 2-Thiocarboxyaldehyde, Trans-2-pentenal, and Glutaraldehyde. At various concentrations, the survival fraction of malignant glioma U87 (MG-U87) cell lines against these MTX derivatives is monitored. These concentrations include 400 μM, 200 μM, 100 μM, 50 μM, 25 μM and 12.5 μM. The survival fraction of malignant glioma cells at different concentration level of MTX derivatives is presented in Fig. 1. This indicates with 12.5 μM concentration all MTX derivatives except MTX-Glutaraldehyde and MTX-2-pentanal compared to MTX-parent compound has better capability to kill MG-U87 cell lines. With 25 μM, 50 μM and 200 μM concentration all MTX derivatives compared to MTX-parent has better capability to kill MG-U87 cell lines. With 100 μM concentration all MTX derivatives except MTX- Glutaraldehyde, MTX-Benzaldehyde and MTX-parent drug has better capability to kill MG-U87 cell lines. With 400 μM concentration all MTX derivatives except MTX-Benzaldehyde, MTX-Glutaraldehyde, MTX-NO2, and MTX-OHNO2 compared to MTX-parent has better capability to kill MG-U87 cell lines.

**Figure 2:**
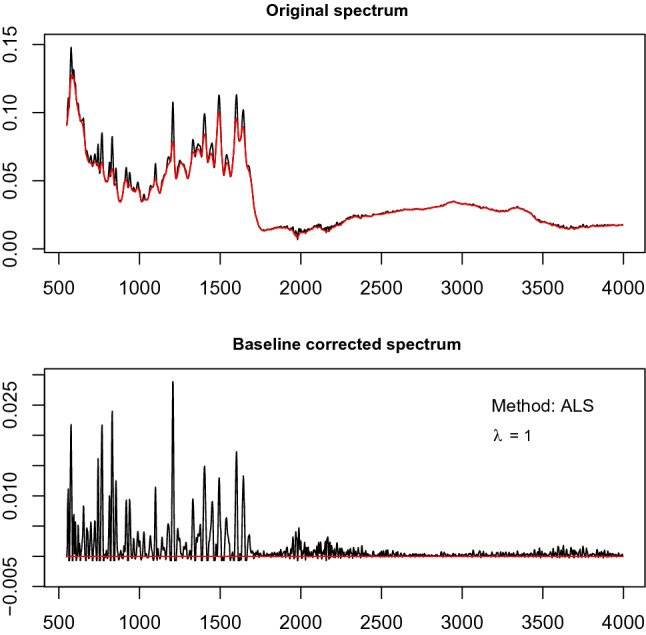
The comparison of original and baseline corrected spectral data is shown here. The base line is corrected using ALS with smoothing parameters (lambda = 1) and weights (w = 0.001).

Te current study aims to predict the survival fraction of malignant glioma cell lines against several concentrations of MTX derivatives through FTIR spectroscopy. The 10 samples of MTX derivatives were subjected to FTIR spectroscopic experiments for this purpose. For the projected mathematical models, the threshold for FTIR spectra should be at 0. For ALS baseline adjustment, we have used algorithms. It is dependent on ALS to select their respective parameters. We also used the objective method for optimum parameter tuning, where combinations of various levels of the parameters are evaluated for the survival fraction by PLS.Cross validation establishes the optimal parameter estimates of ALS. For estimating the survival fraction at different stages of concentration, smoothing parameters $\theta =1$ and wights $w=0.001$ tend to optimally correct the spectral basis of the MTX derivatives. The relation between the continuum of MTX derivatives initial and baseline corrected is seen in Fig. 2. After the baseline correction, the survival fraction of MTX derivatives $Y_{10 \times 6}$ is modeled with spectroscopic data matrix $X_{10 \times 1676}$. As prepossessing, the data matrix was scaled with mean zero and variance 1.

**Figure 3:**
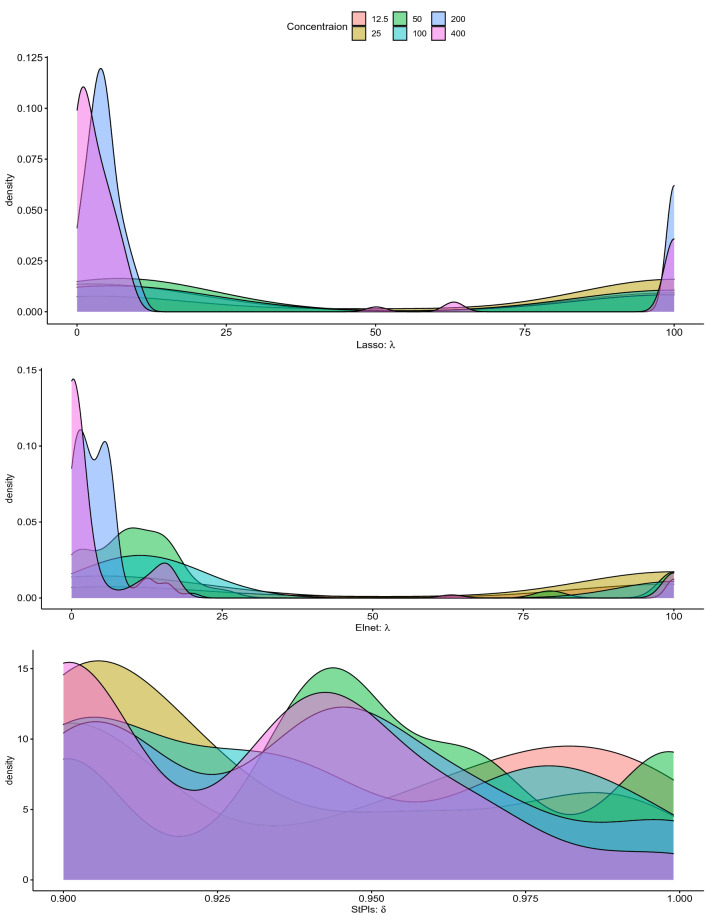
A comparison of the parameters of the fitted model is presented.

**Figure 4:**
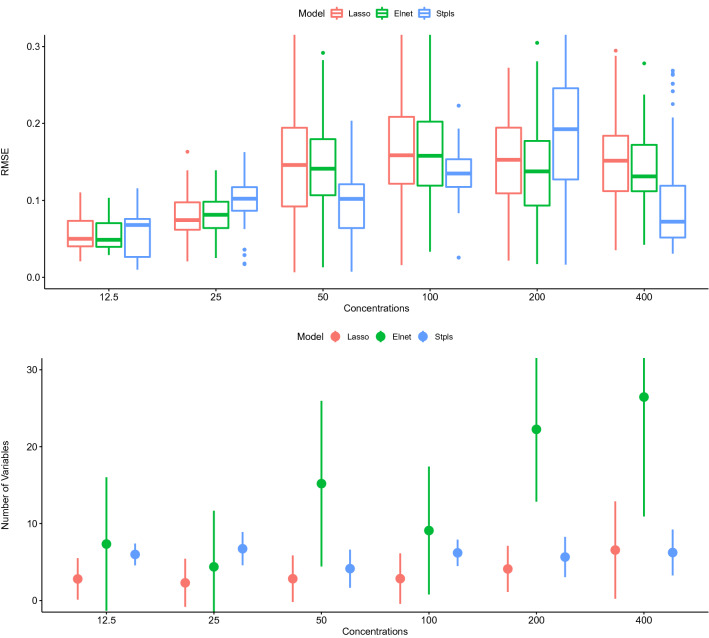
In the upper and lower panels, the distribution of RMSE computed from test data and the distribution of the number of selected variables through Lasso, Elnet and StPLS over all concentration levels is presented.

**Table 1:** The analysis of variance results indicating the significance of concentrations, models and their interactions for explaining the variation in RMSE over test data is presented.

Factors	Df	Sum Sq	Mean Sq	F-value	p-value
Concentration	5	2.65	0.53	66.98	$\le$ 0.001
Model	2	0.07	0.03	4.41	0.012
Concentration:model	10	0.30	0.03	3.80	$\le$ 0.001
Residuals	1782	14.13	0.01		

We use StPLS, Lasso and Elnet for the prediction of the survival fraction of MG-U87 against MTX derivatives at different concentrations. RMSE over the test data is used to measure the prediction performance of considered methods. We have randomly divided samples into test and training data for model building and comparison of the predicted model. Although test samples are used for model comparison or evaluation , training data fits the model. There is random bias. In order to remove this bias, the above data validation and calibration process will be replicated within 100 runs. The distribution of optimal parameter estimates for Lasso, Elnet and StPls obtained against concentration level is shown in Fig. 3. The calculated ideal median Lasso $\theta$ is 3.98, 100, 10, 10, 5 and 3.16 respectively for the MTX 12.5, 25 , 50, 100, 200 and 400 concentrations. The calculated maximum Elnet $\theta$ median for MTX concentrations of 12.5, 25, 50 , 100 , 200, and 400 is 7.94, 100.00, 11.29, 12.59, 3.98 and 0.23 respectively. Similarly, the approximate optimum StPls $\delta$ median is 0.96, 0.92, 0.94, 0.93, 0.94 and 0.93 respectively for the MTX 12.5, 25 , 50, 100, 200, and 400 concentrations.

Lasso, Elnet and StPls are designed to predict the survival fraction at each stage of the MTX concentration using the above approximate parameters. The validated prediction capabilities that are RMSE standardised over the test data are calculated in each sprint. The analysis of variance results indicating the significance of concentrations, models and their interactions for explaining the variation in RMSE over test data is presented in Table 1. It appears on the RMSE computed from test data is significantly varying with the change of models (p = 0.012), with the change of concentrations levels (p $\le 0.001$) and with the change of combination of models and concentration level (p $\le 0.001$). In the upper panel of Fig. 4, the distribution of validated RMSE of all models over all concentrations is presented. The survival fraction of MTX derivatives with 12.5% concentrations seems to be reasonably expected by Lasso and Elnet. The survival fraction of MTX derivatives with a 25% concentration is better estimated by Lasso. The survival fraction of MTX derivatives with concentrations of 50%, 100% and 400% is better estimated by StPls. With 200% concentrations, Elnet better estimates the survival fraction of MTX derivatives. The distribution in the lower panel of Fig. 4 of the number of selected variables (wavenumbers) of all models for all concentrations is provided. It seems that Lasso uses the least number of variables at all concentrations in estimating the survival fraction of MTX derivatives. Elnet uses the maximum number of variables to estimate the survival fraction of MTX derivatives at all concentrations except 25%, where StPls uses the maximum number of variables.

**Figure 5:**
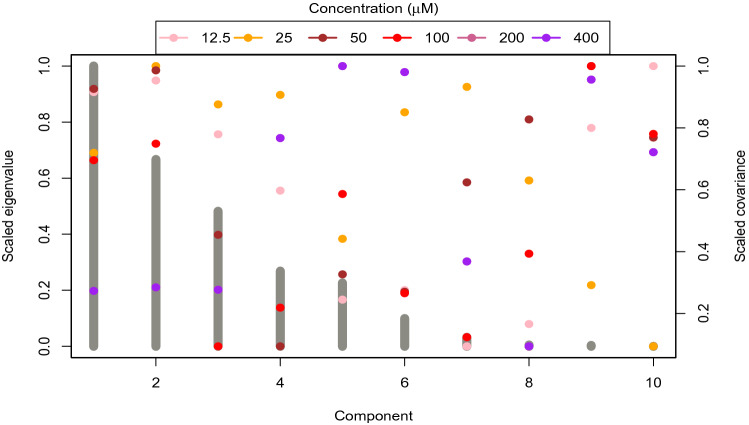
Data properties for the MTX derivative survival fraction as well as spectral data are provided. The bars represent the spectral eigenvalues of ionic fluids (scaled by the largest). The dots show the relationship between the main components and the survival fraction of MTX derivatives at different concentrations (scaled by the largest).

Two model streams exist for prediction purposes, one uses iterative loading based method (StPLS) and others are based on penalised linear models (Lasso and Elnet). The sample covariance matrix’s own value structure of the explanatory matrix is then used to describe the model prediction action’s characteristics and the covariance between the main components and the response response^20,33^. If there are unrelated components with large eigenvalues, the forecast may be worse. As a result, poor prediction of variable selection methods based on PLS is predicted. Figure 5 depicts the data characteristics of the survival fraction of MTX derivatives and spectral data. The bars reflect the ionic fluids’ spectral eigenvalue (scaled by the largest). The dots represent the covariance at different concentrations (scaled by the largest) between the main components and the survival fraction of MTX derivatives. This implies that between-variable dependencies are poor in the spectral data. The data heterogeneity has to be clarified by a large number of latent elements, as we have a limited number of MTX derivatives, making it impossible for the PLS-based model to provide a better estimate of the survival fraction. We observe different behaviour of covariances between the main variable and survival fraction separately from the behaviour of eigenvalues. The own values and covariance are not aligned for relevant components with 12.5%, 25% and 200% concentrations, so a large number of PLS components are recommended for better prediction at these concentrations, which is therefore impossible to achieve here in predicting survival fraction, Lasso or Elnet outperforms.

**Figure 6:**
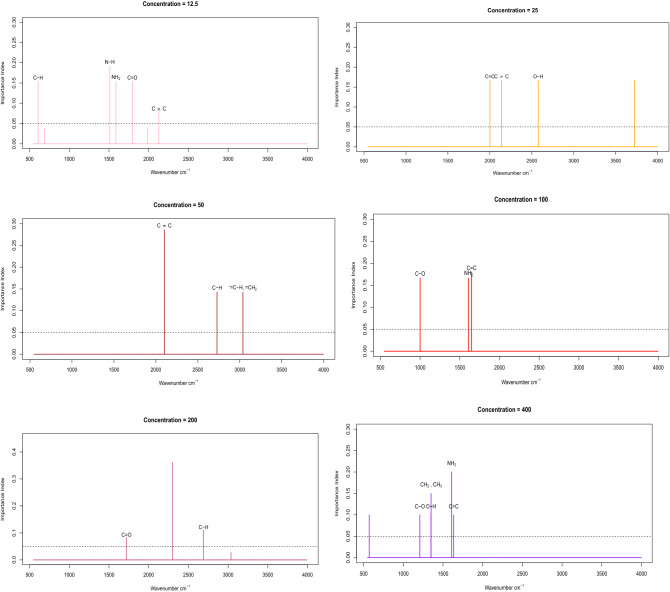
Influential wavenumbers are picked at various concentration levels by the optimal model calculated as shown. Along with a powerful functional group, the relevant regression coefficients of the best suited model are shown.

Influential wavenumbers are chosen by the optimum model defined in Fig. 4, which is seen in Fig. 6 at various concentration levels. Along with an influential functional compound, the strong regression coefficients of the best fitting model are shown. It seems that the prominent wavenumber corresponds to C–H, N–H, N–H2, C=O, C$\equiv$C for prediction of the MTX derivative survival fraction toward 12.5% concentrations. The prominent wavenumber corresponds to C–O, C$\equiv$C, O–H for calculation of the MTX derivative survival fraction against 25% concentrations. The prominent wavenumber corresponds to C–H, =CH2, C=O and C$\equiv$C for the prediction of the MTX derivatives survival fraction toward 50% concentrations. The prominent wavenumber corresponds to C–O, C$\equiv$C and N–H2 for prediction of the survival fraction of the MTX derivatives against 100% concentrations. The prominent wavenumber refers to C–H and C–H for the estimation of the MTX derivative survival fraction against 200% concentrations. The influential wavenumber corresponds to C–O, C$\equiv$C, N–H2, O–H and CH 2, CH3 for prediction of the MTX derivative survival fraction against 400% concentrations.

### Computations

For computations, modeling and figures R software is used^34^. For baseline correction R package ’baseline’^35^ and for model fitting R packages ’glmnet’ and ’plsVarSel’^36,37^ are used.

## Conclusions

Via FTIR spectroscopy by StPLS, Lasso and Elnet, the study predicts the survival fraction of malignant glioma cells against many concentrations of methotrexate derivatives. ASL works well for base-line correction. The survival fraction prediction capabilities are linked with covariance between the survival fraction and spectrum data. Moreover Influential wavenumbers are picked by Lasso at concentrations of 12.5% and 25%, by StPls at concentrations of 50%, 100% and 400%, and by Elnet at concentrations of 200% along with influential functional compounds.

## Supplementary Information

Supplementary Information.
